# Identification of fecal microbiome signatures associated with longevity through 16S rRNA sequencing in different age groups in China

**DOI:** 10.1007/s00253-026-13752-x

**Published:** 2026-02-16

**Authors:** Yuexia Wang, Jie Liu, Wenjing Wang, Gefang Xu, Ying Lin, Pengxia Song, Xingchen Zhou, Kunhua Zheng

**Affiliations:** 1 People’s Hospital of Kaihua, 59 Fenghuang Middle Road, Quzhou, 324300 PR China; 2 Medical School, Quzhou College of Technology, 18 Jiangyuan Road, Quzhou, 324000 PR China; 3 College of Chemical and Biological Engineering, Zhejiang University, 866 Yuhangtang Road, Hangzhou, 310058 PR China; 4 Division of (Bio) Pharmaceutics, Institute of Zhejiang University-Quzhou, 99 Zheda Road, Quzhou, 324000 PR China

**Keywords:** Aging, Gut microbiota, 16S rRNA sequencing, Longevity

## Abstract

**Abstract:**

The gut microbiota plays a key role in aging and longevity. Therefore, identifying longevity-associated microbes in healthy and long-lived individuals and elucidating the molecular mechanisms through which they influence longevity are essential steps toward developing effective anti-aging interventions. In this study, we performed 16S rRNA sequencing on 301 fecal samples collected across three age groups. Long-lived individuals (≥ 90 years) had more diverse gut microbiota than typical older individuals (60–89 years), with diversity comparable to that of younger adults (45–59 years). Compared with typical older individuals, long-lived individuals exhibited a marked increase in the relative abundance of *Bacteroidota* and *Akkermansia*, accompanied by a decreased abundance of *Prevotella_9* and *Megamonas*. Additionally, the microbiota from this age group showed significant enrichment in unsaturated fatty acid metabolism, ketone body synthesis and degradation, and tryptophan metabolism, suggesting that differences in microbiota composition and function may contribute to longevity. Finally, we developed a qPCR-based method to detect differentially abundant microbiota and established a classification model capable of distinguishing between age groups. In conclusion, the unique composition and function of the gut microbiota in long-lived individuals offer insights for identifying methods and targets for anti-aging interventions.

**Key points:**

• *Long-lived individuals exhibited a marked increase in Bacteroidota and Akkermansia*

• *Long-lived individuals exhibited enrichment in unsaturated fatty acid metabolism*

• *We developed a qPCR-based method to detect differentially abundant microbiota.*

## Introduction

According to the National Bureau of Statistics, China entered an era of population aging in 2013. Over the following 6 years, the proportion of individuals aged ≥ 60 years increased by 3.2% (51 million individuals), while those aged ≥ 65 years increased by 2.9% (44 million individuals), indicating a progressively pronounced trend of population aging (Xiang and Wang 2021). Additionally, although per capita life expectancy in China is 76.4 years, the average healthy life expectancy is only 68.7 years, indicating that older individuals may spend approximately 8 years living with disease. This trend is further supported by the growing prevalence of chronic conditions and the increasing number of older adults with functional impairments (Wang 2019), placing a substantial burden on families, society, and the nation in terms of geriatric care. Therefore, identifying determinants of healthy longevity and developing targeted interventions and guidelines are urgently needed to promote healthy aging in China.

The gut microbiota is a key factor associated with healthy longevity (Ghosh et al. 2022). Compared with healthy individuals, older adults and those in poor health exhibit decreased microbial diversity and increased proportions of pathogenic microbes. The composition of the “aging microbiota” changes with age, which not only adversely impacts gastrointestinal digestion and absorption but is also associated with cardiovascular, immune, neurological, and respiratory disorders (Haran and McCormick 2021). After the age of 80, the gut microbiota of centenarians (long-lived population) has been shown to progressively evolve into a distinct composition (Wilmanski et al. 2021), characterized by a decreased abundance of *Faecalibacterium* and *Prevotella* and increased abundance of *Escherichia*, *Akkermansia*, *Clostridium*, and *Collinsella*, compared with the typical gut microbiota of older adults (Kim et al. 2019). Furthermore, this long-lived population reportedly exhibits greater microbial diversity and richness compared with the younger adult population (Kong et al. 2016), suggesting that a higher abundance of beneficial microbes and greater diversity and richness may be key contributors to human longevity and health (Kong et al. 2019).

Accumulated evidence suggests that the gut microbiota influences aging and longevity primarily by regulating inflammatory and metabolic pathways (Buford 2017; Haran and McCormick 2021). These microbes play a critical role in shaping the human immune system (Schluter et al. 2020), with their dysregulation accelerating inflammatory aging, leading to the development of age-related diseases. The gut microbiota of long-lived individuals has been reported to produce secondary bile acids that modulate immune cells and reduce the risk of pathogen infection, thereby contributing to the maintenance of intestinal homeostasis (Sato et al. 2021). Moreover, the gut microbiota of long-lived individuals is better adapted to xenobiotic degradation and can modulate metabolic pathways associated with carbohydrate, amino acid, and lipid metabolism (Rampelli et al. 2020). Notably, the potential of gut microbiota modulation in extending lifespan has been extensively reported (Barcena et al. 2019). Approaches such as probiotic or prebiotic supplementation and microbiota transplantation have been shown to promote the production of beneficial metabolites, enhance the intestinal microenvironment, increase microbial diversity, and decrease inflammation, thereby contributing to delayed aging (Westfall et al. 2018).

In the current study, we collected fecal samples from individuals across three age groups (e.g., ≥ 90 years, 60‒89 years, and 45‒59 years), all of whom were free of any major diseases. Microbial composition and relative abundance were assessed using 16S rRNA sequencing and quantitative PCR (qPCR) to characterize the microbiota profiles of these age groups in the region. By comparing results across age groups, we identified microbial species potentially associated with healthy longevity and constructed a classification model for distinguishing different age groups. Additionally, the mechanisms through which the gut microbiota influence longevity were investigated via functional analysis of 16S rRNA sequencing data.

## Materials and methods

### Ethics approval and informed consent

This study was approved by the Ethics Committee of People’s Hospital of Kaihua (No. 2020–KT–050). All participants signed an informed consent form prior to donating fecal samples.

### Participant information and sample collection

Participants were community-dwelling adults and permanent residents in Kaihua County of Quzhou City (Zhejiang Province, China), which was recognized as “World Longevity-City” by the International Natural Medicine Association. Health-related information was collected from the participants using an in-person questionnaire, covering questions about living conditions, demographics, disease history, dietary habits, medication use, and lifestyle. In addition, researchers collected information regarding hematologic and clinical chemistry test results. Participants were excluded if they had substantial cognitive impairment, severe chronic diseases (diabetes or neurodegenerative diseases), malignant tumors, or acute infectious diseases. In total, 301 fecal samples were collected from individuals across different ages: *n* = 103, aged ≥ 90 years (longevity group); *n* = 100, aged 60–89 years (older group); and *n* = 98, aged 45–59 years (young group). Stool samples were collected by the participants using a specialized fecal specimen collection tube (TS010-5; Genstone Biotech, Beijing, China) and immediately stored at −80 °C for further analysis.

### 16S rRNA sequencing and data processing

DNA from different samples was extracted using the CTAB (hexadecyltrimethylammonium bromide) according to manufacturer’s instructions. Samples were sequenced on an Illumina NovaSeq platform (San Diego, CA, USA) according to the manufacturer’s recommendations, provided by LC-Bio Technologies Co., Ltd (Hangzhou, Zhejiang Province, China), and then the raw 16S rRNA sequencing data was submitted to SRA database which was NIH’s (National Institutes of Health) archive of high-throughput sequencing data (https://www.ncbi.nlm.nih.gov/sra). Paired-end reads were merged using FLASH (v1.2.8) (Magoc and Salzberg 2011). Quality filtering on the raw reads were performed under specific filtering conditions to obtain the high-quality clean tags according to the fqtrim (v0.94) (https://ccb.jhu.edu/software/fqtrim/index.shtml). Chimeric sequences were filtered using Vsearch software (v2.3.4) (Rognes et al. 2016). After dereplication using DADA2 (QIIME2, 2019.7) (Callahan et al. 2016), we obtained feature table and feature sequence. Alpha diversity and beta diversity were calculated by normalized to the same sequences randomly. Then according to SILVA classifier (release 138, https://www.arb-silva.de/documentation/release-138) (Quast et al. 2013; Yilmaz et al. 2014), feature abundance was normalized using relative abundance of each sample. Alpha diversity is applied in analyzing complexity of species diversity for a sample through five indices, namely Chao1, Observed species, Goods coverage, Shannon, and Simpson, and all this indices in our samples were calculated with QIIME2 (Bolyen et al. 2019). Beta diversity were calculated by QIIME2, and the graphs were drawn by R package (v3.4.4) (R Core Team 2018). Blast (Altschul et al. 1990) was used for sequence alignment, and the feature sequences were annotated with SILVA database for each representative sequence.

### Composition and differential analyses

Based on the feature annotations and corresponding abundance tables for each sample, taxonomic abundance profiles were generated at the kingdom, phylum, class, order, family, genus, and species levels. Using the taxonomic profiles, microbial composition and differential abundance analyses were then conducted across the age groups.

### Functional and differential analyses

Functional prediction of microbial communities was performed using PICRUSt2 (picrust2.2.0b) (Douglas et al. 2018), which infers gene family abundances by mapping ASVs (Amplicon Sequence Variants) to known functional profiles. Based on PICRUSt2 predictions, functional annotations for gene families were obtained across multiple databases, including COG (https://www.ncbi.nlm.nih.gov/research/cog), EC (https://enzyme.expasy.org/index.html), KO (https://www.kegg.jp/kegg/ko.html), PFAM (http://pfam.xfam.org/), and TIGRFAM (https://tigrfams.jcvi.org/cgi-bin/index.cgi). Microbiome phenotypic traits, such as potential pathogenicity and stress tolerance, were further predicted using BugBase (https://github.com/knights-lab/BugBase) (Duan and Li 2023). Differences in predicted functional pathways among age groups were identified using STAMP (Parks et al. 2014).

### qPCR validation

#### DNA extraction

DNA was extracted from fecal samples using the Soil/Fecal DNA Small Volume Extraction Kit (TD601-50; Genstone Biotech, Beijing, China). Briefly, samples were lysed using a lysis buffer and centrifuged to remove the supernatant. The resulting pellet was then treated with genomic DNA lysis buffer and passed through a purification column to isolate the DNA. Multiple wash steps were performed to remove impurities, including potential PCR inhibitors, followed by elution of purified DNA. DNA concentration was quantified using a Qubit® 3.0 fluorometer of Thermo Fisher Scientific (Waltham, MA, USA).

#### qPCR

Using bioinformatics tools, five sets of genus-specific primers were designed and synthesized by Tsingke Biotechnology (Beijing, China). The primer sequences were as follows: Alistipes-F: AGTCGGCTGCGGTATATGC; Alistipes-R: TGCGACACCCATCACCTTC; Akkermansia-F: ACATGCACATCGACGGCA; Akkermansia-R: TGCGTGTCTTCATGTCCCC; Klebsiella-F: TCACTGCCAGTTCGTGCT; Klebsiella-R: TCGAACGGTTACTGCGCT; Megamonas-F: GTCAGGTTGTGGAAGCGAAAC; Megamonas-R: ACGTTGAATATCCATCTGGGC; Prevotella-F: AACGGACTGCAGGTTGGTG; and Prevotella-R: AGCCGAACGAACGAGCAA. The relative abundance of the five differential genera was quantified using the LightCycler 96 real-time PCR system (Roche, Mannheim, BW, Germany). qPCR was performed in a 20 μL reaction mixture containing 1 μL of template DNA, 1 μL each of forward and reverse primers, and 10 μL of FastReal qPCR PreMix (SYBR Green; FF240124; Tiangen Biotech, Beijing, China). The qPCR protocol was as follows: 95 °C for 2 min, followed by 40 cycles of 95 °C for 5 s and 59 °C for 30 s.

### Statistical analysis

All statistical analyses were performed using R software (version 3.4.4) (R Core Team 2018). Microbial abundance was compared using the Mann-Whitney *U* test for pairwise comparisons between two groups with biological replicates, and the Kruskal-Wallis test for comparisons across multiple groups. The results of the STAMP analysis are presented for the top 30 functions showing significant differences (*p* < 0.05), based on pairwise comparisons using the two-sided *t*-test.

## Results

### Compositional differences in gut microbiota across the three age groups

The raw 16S rRNA sequencing data have been submitted to the SRA database with BioProject ID PRJNA1320995. Analysis of α-diversity revealed that all six indices indicated higher microbial diversity in the longevity group than in the older group, with no significant differences observed when compared with the young group. Notably, two indices (Chao1 and Observed OTUs) revealed significantly lower diversity in the older group than in the young group. Regarding β-diversity, analysis of similarities (ANOSIM) revealed that between-group differences (among two or more groups) were greater than within-group differences, indicating statistically significant separation among groups (*p* < 0.05).

*Firmicutes*, *Bacteroidota*, and *Proteobacteria* were identified as the three most abundant phyla. The relative abundance of *Bacteroidota* was significantly higher in both the young and longevity groups than in the older group, with no significant difference observed between the young and longevity groups. The abundance of *Proteobacteria* varied substantially across the three age groups, exhibiting an increasing trend with age. In contrast, the abundance of *Firmicutes* was comparable across all age groups (Table 1). At the genus level, the longevity group exhibited significantly higher relative abundances of *Klebsiella*, *Akkermansia*, and *UCG-002*, and lower abundances of *Prevotella_9*, *Megamonas*, and *Agathobacter* compared with the older group (Table 2). Conversely, the older group demonstrated significantly higher abundances of *Prevotella_9*, *Escherichia–Shigella,* and *Fusobacterium*, and lower abundances of *Arthrobacter*, *Lachnospira*, and *Megamonas* relative to the young group.

**Table 1:** Differences in abundance of major phyla across age groups

Phylum	Longevity vs. older			Longevity vs. young			Older vs. young		
	Log2FC	*P*-value	Regulation	Log2FC	*P*-value	Regulation	Log2FC	*P*-value	Regulation
*p__Firmicutes*	0.06	0.4002	Up	− 0.07	0.3591	Down	** − **0.12	0.1247	Down
*p__Bacteroidota*	** − 0.3**	**0.0153**	**Down**	0.08	0.4757	Up	**0.38**	**0.0024**	**Up**
*p__Proteobacteria*	**0.37**	**0.0138**	**Up**	**1.58**	**0**	**Up**	**1.21**	**0**	**Up**
*p__Verrucomicrobiota*	**2.19**	**0.0107**	**Up**	1.64	0.1137	Up	** − **0.55	0.2447	Down
*p__Fusobacteriota*	− 1.62	0.233	Down	** − 0.55**	**0.0082**	**Down**	**1.07**	**0.0013**	**Up**
*p__Actinobacteriota*	− 0.03	0.1126	Down	** − 3.1**	**0**	**Down**	** − 3.07**	**0**	**Down**

Bold indicates significant differences with *p* value < 0.05

**Table 2:** Differences in abundance of genus between longevity group and older group

Genus	Log2FC	Wilcox-test, *p*-value	Regulation	Mean	Mean_Longevity	Mean_Older
*g__Prevotella_9*	− 0.98	0.01	Down	9.20	6.23	12.25
*g__Klebsiella*	0.83	0.00	Up	2.35	3.00	1.69
*g__Akkermansia*	2.22	0.02	Up	2.19	3.57	0.77
*g__Megamonas*	− 1.83	0.00	Down	1.56	0.69	2.45
*g__UCG-002*	1.01	0.00	Up	1.41	1.88	0.93
*g__Firmicutes_unclassified*	0.48	0.05	Up	1.36	1.59	1.13
*g__Lachnospiraceae_unclassified*	0.53	0.03	Up	1.33	1.57	1.09
*g__Alistipes*	1.09	0	Up	1.15	1.55	0.73
*g__Agathobacter*	− 0.15	0.00	Down	1.08	1.03	1.14
*g__UCG-005*	1.60	0.00	Up	0.41	0.61	0.20
*g__Muribaculaceae_unclassified*	0.54	0.00	Up	0.39	0.46	0.31
*g__Hungatella*	1.20	0.00	Up	0.31	0.43	0.19
*g__NK4A214_group*	0.99	0.02	Up	0.29	0.38	0.19
*g__Oscillibacter*	0.94	0.00	Up	0.26	0.34	0.18
*g__Desulfovibrio*	1.63	0.00	Up	0.26	0.39	0.13
*g__Erysipelotrichaceae_UCG-003*	− 0.91	0.01	Down	0.25	0.17	0.33
*g__Ruminococcaceae_unclassified*	1.80	0.00	Up	0.24	0.37	0.11
*g__Sphingomonas*	1.99	0.00	Up	0.22	0.34	0.09
*g__Bilophila*	0.69	0.01	Up	0.21	0.26	0.16
*g__Butyricimonas*	1.67	0.00	Up	0.20	0.30	0.09

The genus with *p*_value < 0.05, and mean abundance ranks in the top 20

### Functional differences in gut microbiota across the three age groups

**Fig. 1:**
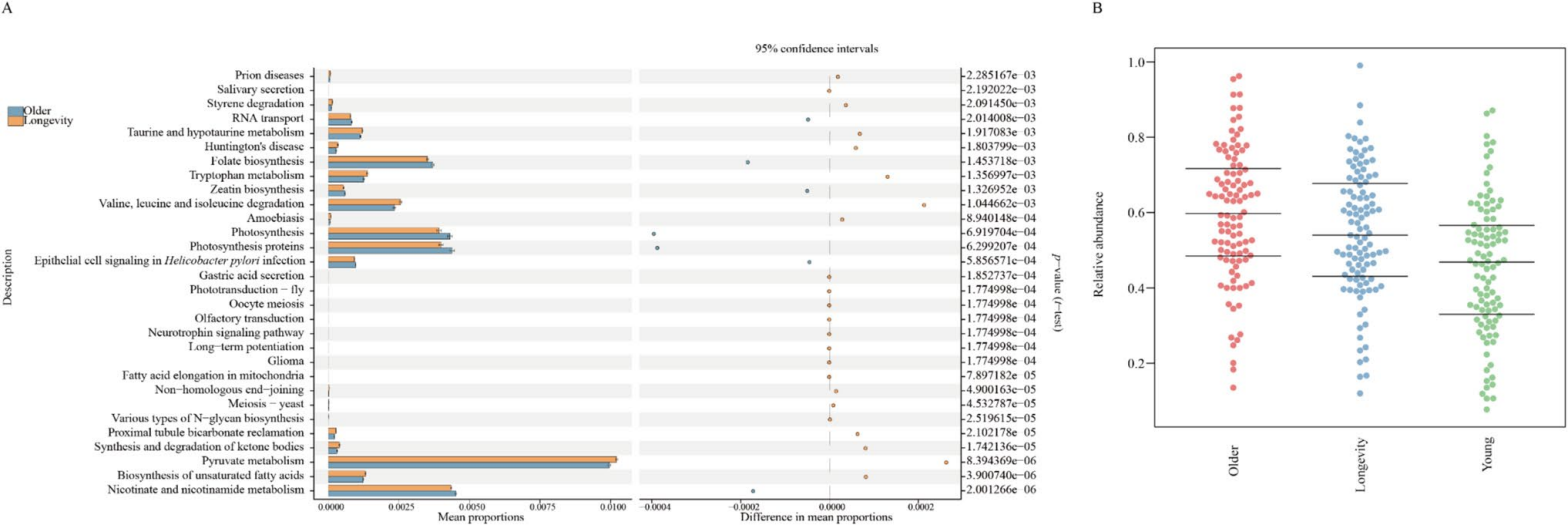
Functional prediction of gut microbiota in different age groups. **A** The top 30 KEGG pathways showing significant differences between longevity group and older groups; **B** predicted microbial pathogenicity across the three age groups (*p* < 0.05). Note: Mean proportions in Fig.  1 A stand for functional category (KEGG pathways) abundances in longevity group and older group respectively. Scatter plot in Fig.  1 A shows statistical difference in the KEGG pathways between two groups and the color of dot indicates which group shows more activities for these pathways. Relative abundance in Fig. 1B stand for abundance of the phenotype (pathogenic)

Functional predictions using PICRUSt2 indicated that the longevity group exhibited significant enrichment in several KEGG level 3 pathways, including biosynthesis of unsaturated fatty acids; pyruvate metabolism; synthesis and degradation of ketone bodies; valine, leucine, and isoleucine degradation; and tryptophan metabolism. In contrast, the longevity group exhibited markedly reduced enrichment in pathways such as nicotinate and nicotinamide metabolism and epithelial cell signaling in *Helicobacter pylori* infection, compared with the older group (Fig. 1A). When comparing the older and young groups, a limited number of pathways, including cell division, pore ion channels, and membrane and intracellular structural molecules, were significantly enriched in the older group. Conversely, the older group demonstrated significantly reduced enrichment in pathways, including glycolysis/gluconeogenesis, pyruvate metabolism, synthesis and degradation of ketone bodies, biosynthesis of unsaturated fatty acids, tyrosine metabolism, glycine, serine, and threonine metabolism, as well as valine, leucine, and isoleucine biosynthesis and degradation. Furthermore, microbial phenotype prediction using BugBase indicated a significantly higher abundance of potentially pathogenic microbes in the older group compared with both the longevity and young groups, respectively, as well as in the longevity group compared with the young group (Fig. 1B).

### Development of a qPCR-based method for detecting longevity-associated microbes

**Fig. 2:**
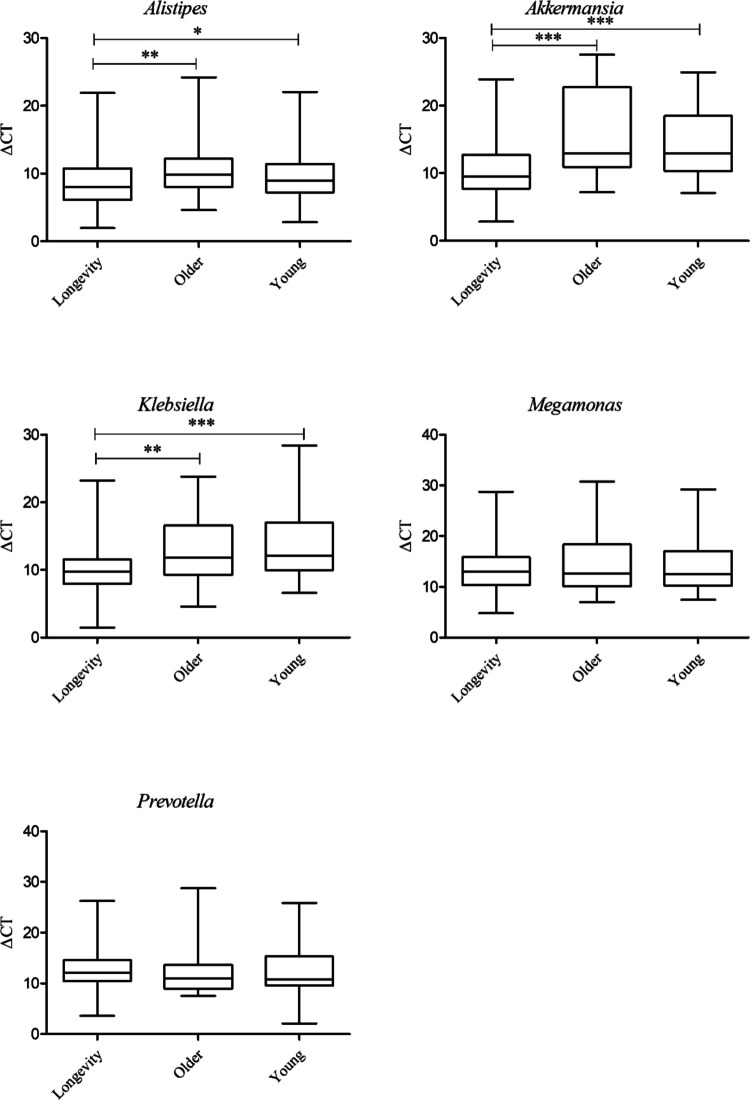
Relative abundance of five genera measured by qPCR in different age groups (△Ct = Ct_Genus_-Ct_control_, the smaller △Ct value corresponds to a higher abundance). Note: “***,” “**,” and “*” stand for *p* value < 0.001, < 0.01, and < 0.05, respectively.

Based on comparative analysis of microbial abundance between the longevity and older groups, five differentially abundant genera (*Alistipes*, *Akkermansia*, *Klebsiella*, *Megamonas*, and *Prevotella*) were selected for qPCR detection using genus-specific primers. qPCR results revealed that the relative abundances of *Alistipes*, *Akkermansia*, and *Klebsiella* were significantly higher in the longevity group compared with both the older and young groups, respectively, with no significant differences observed between the older and young groups. In contrast, the abundances of *Megamonas* and *Prevotella* were comparable across all three age groups (Fig. 2). These results were largely consistent with the 16S rRNA sequencing data for the first three genera, with slight discrepancies observed for *Megamonas* and *Prevotella*.

### Construction of a multiple logistic regression classification model

**Fig. 3:**
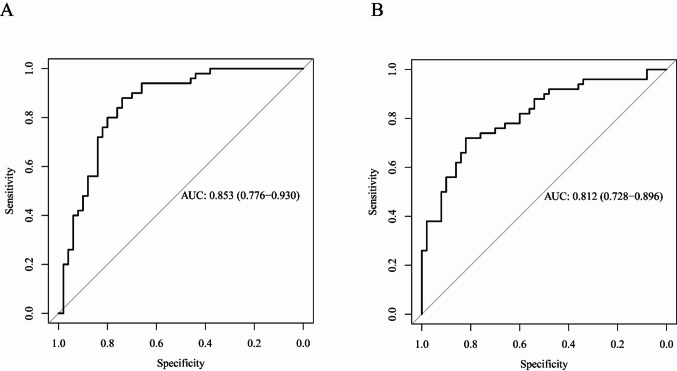
Receiver operating characteristic (ROC) curves of the classification model for distinguishing different age groups. **A** Longevity vs. older; **B** Longevity vs. young. Note: AUC stands for area under the curve. It’s a key metric used to evaluate the performance of a binary classification model

Binary logistic regression models were constructed using the relative abundances of differentially abundant genera, calculated as ΔCt (**△**Ct = Ct_Genus_-Ct_control_). The final model based on four genera (*Akkermansia*, *Klebsiella*, *Megamonas*, and *Prevotella*) effectively distinguished between the longevity and older groups, achieving a sensitivity of 82.0% (95% confidence interval [CI] 69.0%–90.5%), a specificity of 76.0% (95% CI 62.5%–85.8%), and an area under the receiver operating characteristic (ROC) curve (AUC) of 0.853 (95% CI 0.776–0.930) (Fig. 3A). A logistic regression model constructed using the relative abundances of three genera (*Akkermansia*, *Klebsiella*, and *Prevotella*) successfully differentiated between the longevity and young groups, with a sensitivity of 78.0% (95% CI 64.6%–87.4%), a specificity of 72.0% (95% CI 58.2%–82.6%), and an AUC of 0.812 (95% CI 0.728–0.896) (Fig. 3B). In contrast, differences in the relative abundances of these five genera were insufficient to effectively distinguish between the older and young groups.

## Discussion

In this study, we demonstrated that long-lived individuals have a more diverse gut microbiota than older individuals, with diversity levels comparable to those observed in younger adults, consistent with previous findings. Significant differences in the relative abundance and predicted functions of several bacterial phyla and genera were identified across three age groups. Finally, we developed a qPCR-based method to detect differentially abundant microbes and constructed a logistic regression classification model capable of distinguishing between age groups. This model may serve as a promising tool for predicting longevity or evaluating healthy aging.

*Bacteroidota* was the most abundant phylum in the gut microbiota, demonstrating significantly higher relative abundance in both the longevity and young groups compared with the older group, with no significant difference between the longevity and young groups. An increased abundance of *Bacteroidota* has been identified as a hallmark of longevity (Pang et al. 2023). At the genus level, the longevity group exhibited significantly higher abundances of *Klebsiella* and *Akkermansia*, and lower abundances of *Prevotella_9* and *Megamonas* compared with the older group. Furthermore, *Akkermansia* may play a role in prolonging longevity (Ioannou et al. 2025; Zeng et al. 2023). Notably, *Akkermansia* spp. stimulate intestinal epithelial cells to secrete more mucus, thereby increasing the thickness of the mucus layer and protecting against the invasion of pathogens and harmful substances (Mo et al. 2024). Additionally, *Akkermansia* spp. help maintain intestinal barrier integrity by upregulating the expression of tight junction proteins such as ZO-1 and occludin (Bian et al. 2019). *Akkermansia* spp. have also been shown to modulate immune responses by influencing immune cell populations (e.g., CD8⁺ T cells) (Ansaldo et al. 2019) and reducing levels of proinflammatory mediators, thereby attenuating both local and systemic inflammation (Zhao et al. 2024). Short-chain fatty acids, such as acetic and propionic acids, produced by *Akkermansia* spp., not only serve as an energy source for intestinal epithelial cells but also ameliorate metabolic disorders by enhancing insulin sensitivity and regulating glucose metabolism (Rodrigues et al. 2022; Yoon et al. 2021). By exerting these synergistic effects, *Akkermansia* spp. play a key role in maintaining the integrity of the intestinal mucosal barrier, supporting a balanced gut microbiota, and promoting overall health (Cani et al. 2022). The association between *Megamonas*, a prominent genus in the gut microbiota of Asian populations, and human diseases remains poorly understood. Preliminary evidence suggests potential associations between inflammatory bowel disease, colorectal cancer, ankylosing spondylitis, obesity, and neurological disorders. However, specific causal relationships and underlying molecular mechanisms warrant further investigation. A reduced relative abundance of *Megamonas* has been reported in long-lived individuals (Ai et al. 2024). Consistently, our earlier study (Liu et al. 2024) and current findings demonstrate a lower abundance of *Megamonas* in the longevity group compared with that in the older group. These findings suggest that *Megamonas* may be detrimental to longevity, although further research is needed to elucidate its biological role. Likewise, *Prevotella* is reportedly less abundant in long-lived populations (Shi et al. 2022), warranting further investigation into its potential impact on healthy aging.

Regarding pathway enrichment, biosynthesis of unsaturated fatty acids, pyruvate metabolism, synthesis and degradation of ketone bodies, valine, leucine, and isoleucine degradation, and tryptophan metabolism were significantly enriched in the longevity group, whereas nicotinate and nicotinamide metabolism and epithelial cell signaling in *H. pylori* infection were less enriched, compared with the older group. Unsaturated fatty acids have been reported to extend lifespan (Aiello et al. 2024; Gao et al. 2024; Liu et al. 2025). Monounsaturated fats have been shown to facilitate weight management and glycemic control, while polyunsaturated fats are associated with a reduced risk of cardiovascular disease. Papsdorf et al. (2023) demonstrated that supplementation with cis-monounsaturated fatty acids, such as oleic acid from olive oil, increased the formation of lipid droplets and peroxisomes, which, in turn, contributed to synergistic anti-aging effects and extended lifespan. Lipid droplets store fatty acids and help reduce lipid peroxidation, whereas peroxisomes support metabolic homeostasis by scavenging free radicals (Papsdorf et al. 2023). Our results revealed that ketone body production and degradation were enhanced in the gut microbiota of long-lived individuals. More recently, the beneficial role of ketone bodies as health-promoting metabolites has received growing attention. Beyond serving as a key energy source under low-carbohydrate conditions, ketone bodies reportedly exert anti-aging effects by promoting mitophagy (Puchalska and Crawford 2017). Further research is needed to explore their specific applications across different age groups to facilitate the development of ketone body-based interventions to promote healthy longevity. Multiple amino acid metabolic pathways were upregulated in the longevity group, including tryptophan metabolism, suggesting a potential role in supporting longevity. For instance, the tryptophan-derived metabolite 5-methoxyindoleacetic acid, which is associated with the *Christensenellaceae R-7* group, has been implicated in delaying aging, extending lifespan, and reducing inflammation (Qiu et al. 2025).

A major goal of longevity-related microbiome research is to develop reliable methods for predicting healthy aging. Early detection of microbial disturbances or dysbiosis in older adults may facilitate targeted interventions that support healthier aging trajectories. In this cross-sectional study, we identified five genera that effectively distinguish long-lived individuals from typical older adults. Accordingly, we developed a qPCR-based detection method and constructed a logistic regression classification model, which identified long-lived individuals with 82.0% sensitivity, 76.0% specificity, and an AUC of 0.853. Future cohort studies will be conducted to validate the predictive performance of this model through long-term follow-up to establish its clinical utility as a tool for predicting healthy aging.

A key limitation of this study is its cross-sectional design, which enables the identification of associations but does not permit causal inference. Although we observed a significantly reduced relative abundance of *Megamonas* in the longevity group compared with other age groups, it remains unclear whether this reduction contributes to longevity or if the physiological characteristics of long-lived individuals result in reduced *Megamonas* abundance.

## Conclusions and implications

In summary, we analyzed fecal samples across different age groups and identified a longevity-associated microbiota profile characterized by increased diversity and richness, a marked increase in the relative abundance of *Bacteroidota* and *Akkermansia*, along with significantly enhanced metabolism of unsaturated fatty acids, tryptophan, and so on. In addition, we developed a qPCR-based method to detect differentially abundant microbiota and established a classification model capable of distinguishing between longevity and older groups. However, further research is needed to elucidate the specific mechanisms and pathways through which these beneficial microbial genera contribute to longevity and delay aging.

## Data Availability

The raw 16S rRNA sequencing data have been submitted to the SRA database with BioProject ID PRJNA1320995.
